# Functional Dentition in Brazilian Adults: An Investigation of Social Determinants of Health (SDH) Using a Multilevel Approach

**DOI:** 10.1371/journal.pone.0148859

**Published:** 2016-02-10

**Authors:** Loliza L. F. H. Chalub, Carolina C. Martins, Raquel C. Ferreira, Andréa M. D. Vargas

**Affiliations:** 1 Department of Community and Preventive Dentistry, School of Dentistry, Federal University of Minas Gerais [Universidade Federal de Minas Gerais], Belo Horizonte, Minas Gerais, Brazil; 2 Department of Pediatric Dentistry and Orthodontics, School of Dentistry, Federal University of Minas Gerais [Universidade Federal de Minas Gerais], Belo Horizonte, Minas Gerais, Brazil; Johns Hopkins Bloomberg School of Public Health, UNITED STATES

## Abstract

**Objectives:**

Estimate the prevalence of functional dentition among Brazilian adults using four different definitions and identify associated factors.

**Methods:**

A cross-sectional study was conducted involving 9564 Brazilian adults aged 35–44 years who participated in the 2010 National Oral Health Survey. Data collection involved oral examinations and the administration of questionnaires. The following definitions were used: 1—WHO Functional Dentition (FDWHO: ≥ 20 teeth present); 2—well-distributed teeth (WDT: ≥ 10 teeth in each arch); 3 –Functional dentition classified by esthetics and occlusion (FD_Class5_: dentitions that sequentially exhibit at least one tooth in each arch, at least 10 teeth in each arch, all maxillary and mandibular anterior teeth, three or four premolar posterior occluding pairs [POPs], and at least one molar POP bilaterally); 4—Functional dentition classified by esthetics, occlusion and periodontal status (FD_Class6_: corresponds to FD_Class5_ with the addition of periodontal status of all sextants in the oral cavity with, at most, shallow pockets and/or clinical attachment level of 5 mm (CPI ≤ 3 and/or CAL ≤ 1). The independent variables were individual factors (gender, self-declared skin color, schooling, monthly household income, age group, self-rated treatment need, dental pain, dental appointment in the previous 12 months and dental services) and contextual factors (Municipal Human Development Index [MHDI]), Gini coefficient, fluoridated water supply and oral health coverage). Multilevel mixed-effect Poisson regression analyses were performed.

**Results:**

The prevalence of functional dentition based on the FDWHO, WDT, FD_Class5_ and FD_Class6_ definitions was 77.9%, 72.9%, 42.6% and 40.3%, respectively. Adults with ≥12 years of schooling and monthly household income from US$ 853 to 2557 had higher prevalence rates of FDWHO (PR: 1.41 and 1.10, respectively), WDT (PR: 1.58 and 1.14, respectively), FD_Class5_ (PR: 2.03 and 1.27, respectively) and FD_Class6_ (PR: 2.15 and 1.35, respectively). These values in the final models were adjusted for gender, self-declared skin color (FD_Class5_), age group, self-rated treatment need (FDWHO, FD_Class5_ and FD_Class6_), dental appointment in the previous 12 months (FDWHO and WDT), dental services (FDWHO and WDT) and contextual factors. A very high MHDI and presence of fluoridated water supply were associated with higher prevalence rates of the four outcomes.

**Conclusions:**

The incorporation of the criteria of new definitions of functional dentition led to a lower prevalence rate among Brazilian adults. Striking individual and contextual inequalities were identified with regard to the four definitions analyzed, which need to be addressed through inter-sector efforts.

## Introduction

Reduced dental arches that preserves basic functions, such as chewing, speaking and esthetics, are characterized as a *functional dentition* (FD). The World Health Organization (WHO) establish that FD is the retention of a natural, esthetic, functional dentition of no less than 20 teeth throughout life with no need for tooth replacement [1]. This definition of FD based on the quantitative WHO criteria (FDWHO) is the most employed in the literature [2–6]. However, since each tooth group performs a different function, the quantitative concept has been questioned, as the mere number of teeth seems to be a simplistic definition in terms of functionality. Thus, Nguyen *et al*. [7] developed a dental functional status classification system with five levels that consider the following requirements: at least one tooth in each arch, at least 10 teeth in each arch, all maxillary and mandibular anterior teeth, three to four posterior occluding pairs (POPs) and at least one molar POP bilaterally. This new definition of FD has been evaluated in different populations in Europe and Southeast Asia [7–9] and has been employed recently in Latin America [10]. The functionality criteria of this system have demonstrated positive impacts on chewing function for both fibrous and pasty foods [11,12] as well as greater satisfaction with mouth [8] and better oral health-related quality of life (OHRQoL) [9]. However, this system does not address periodontal status, which is important to the definition of FD, as the loss of periodontal tissue exerts a negative impact on chewing function [13].

Despite the limitation of the FDWHO quantitative concept, the global goal of increasing the number of individuals with FD [2] is important and should be incorporated into public policies. Since recent findings demonstrate that the natural teeth ensure greater satisfaction and OHRQoL in comparison to prosthetic replacements [8,9], there is the need for efforts directed at achieving this goal in the adult population.

FD has been selected as the outcome variable in some studies [3–6]. These studies have found positive associations between FD and the male gender, better levels of income and schooling, a visit to the dentist in previous 12 months, a younger age [3–6], the habit of not smoking and the use of dental floss [6] (individual factors) as well as higher mean level of municipal schooling and the presence of a fluoridated water supply [3] (contextual factors).

Few studies have evaluated the relationship between Social Determinants of Health (SDH) and FD in adults [3–6]. Such an analysis is relevant, since knowing the factors associated with FD at an earlier age may help maintain a functional dentition at an older age. Moreover, to the best of our knowledge, no previous study has compared the association between SDH and different definitions of FD. Thus, the aim of the present study was to estimate the prevalence of functional dentition in Brazilian adults aged 35 to 44 years using four different definitions and identify associated individual and contextual factors.

## Materials and Methods

### Study design and sample

The data employed in the present cross-sectional study were taken from the 2010 National Oral Health Survey (NOHS) conducted by the Brazilian Ministry of Health (BMH) in the five regions of the country [14]. The division of Brazil into five large regions (north, northeast, central west, southeast and south) was determined by the Brazilian Institute of Geography and Statistics (IBGE) and has been used in epidemiological studies with a national scope. Thus, these regions were adopted in the sampling project, along with the capitals of the 27 Brazilian states, including the Federal District, which totaled 32 domains formed by 177 municipalities (27 capitals and 30 municipalities in each region). The sample was obtained through the random selection of municipalities and census sectors, configuring multi-stage cluster sampling with probability proportional to size [15]. Detailed information on the method employed is found in others publications [15,16].

For the 35-to-44-year-old age group used in the present study, the calculation of the sample size was based on the mean number of decayed, missing and filled teeth (DMFT) found in each domain on the national survey conducted in 2003 [17]. The sample size was increased to compensate for a possible 20% loss rate and a design effect of 2 [15].

### Data collection

Data collection involved oral examinations to determine the prevalence and severity of the main oral health conditions and the administration of questionnaires addressing demographic characteristics, socioeconomic status, perceptions regarding oral health and the use of dental services. The field teams were formed by an examiner (dentist) and annotator who had undergone 32 hours of training workshops. Consensus calibration was adopted to calculate the level of agreement between each examiner and the results obtained by consensus of the team. Kappa coefficients were calculated for each examiner and condition studied, with 0.65 established as the minimum acceptable value [16].

The oral examinations were performed following the guidelines of the WHO manual for epidemiological studies [18], using the DMFT index, the Community Periodontal Index (CPI) and the Clinical Attachment Level (CAL) for the determination of tooth status and periodontal status, respectively. Among all the oral data collected, only the DMFT index, the number of teeth (including 3rd molars), number of POPs and the CPI/CAL codes of the sextants were considered in the present study. The total number of teeth was determined by the number of teeth present, excluding codes 4 and 5 (missing) and 8 (unerupted) of the DMFT index. A POP was defined as a pair of antagonist posterior teeth on each side of the mouth, such as the pairs formed by teeth 16 and 46 and teeth 26 and 36. Periodontal status was determined by the highest CPI and CAL codes encountered among the sextants. Satisfactory periodontal status was defined as follows: all sextants in the oral cavity with, at most, shallow pockets and/or clinical attachment level of 5 mm (CPI ≤ 3 and/or CAL ≤ 1).

### Variables

The dependent variables were defined as four oral health outcomes, which were based on the original variables available in the databanks (DMFT index, CPI and CAL), as follows:

1. **Outcome 1 –WHO functional dentition (FDWHO):** presence of 20 or more teeth in the mouth;
2. **Outcome 2 –Well-distributed teeth (WDT):** presence of at least 10 teeth in each arch;
3. **Outcome 3 –Functional dentition classified by esthetics and occlusion (FD**_**Class5**_**):** dentitions that sequentially exhibit at least one tooth in each arch, at least 10 teeth in each arch, all maxillary and mandibular anterior teeth, three or four premolar POPs, and at least one molar POP bilaterally.
4. **Outcome 4—Functional dentition classified by esthetics, occlusion and periodontal status (FD**_**Class6**_**):** corresponds to FD_Class5_ with the addition of periodontal status of all sextants in the oral cavity with, at most, shallow pockets and/or clinical attachment level of 5 mm (CPI ≤ 3 and/or CAL ≤ 1). Further details on the outcomes have been described by Chalub *et al*. [10].

The independent variables were composed of individual (intermediate SDH) and contextual (structural and intermediate SDH) factors associated with health (Table 1). The selection of the variables was based on one of the most widely known conceptual theoretical models of SDH proposed by Solar and Irwin [19], which was adopted by the WHO commission on SDH [20] (S1 Fig, Final form of the Commission on Social Determinants of Health [CSDH] conceptual framework). Structural SDH constitute social, political and economic factors that improve one’s socioeconomic position (the 2010 Municipal Human Development Index and Gini coefficient are used to represent this category), which gives rise to specific intermediate health determinants (Table 1). The main intermediate SDH are material circumstances as well as behavioral, biological and psychosocial factors [19]. The contextual and individual variables selected to represent the intermediate determinants were fluoridated water supply, oral health coverage, gender, self-declared skin color, schooling, monthly household income, age group, self-rated treatment need, dental pain, dental appointment in the previous 12 months and dental services. The importance of studying these factors in the field of dentistry became all-the-more evident with the adaptation of the SDH model to oral health by Watt and Sheiham [21] to address inequalities so that the healthcare sector does not keep repeating the same pattern, namely, only focusing on behavioral changes [19,21]. The nine variables that compose the individual factors were acquired using questionnaires during the data collection process. Gender and self-declared skin color were not used as biological makers, but potentially reflect socio-demographic position, which can exert an influence on the distribution of oral health [22]. Schooling and monthly household income are representative of socioeconomic position and respectively reflect the consequences of life course regarding access to education and surrounding conditions. There variables were collected in a continuous quantitative format and categorized in a similar manner to the pattern employed in the literature [4,22–24] (Table 1).

**Table 1:** Independent variables according to analysis level and to Social Determinants of Health (SDH) categories.

Level	Classification	Variable	Description and categories
1^st^ level—Individual	Intermediate SDH	Gender	Sex of individual (female/male)
		Self-declared skin color	Self-declared skin color (black/white, yellow, brown, indigenous)
		Schooling	Completed years of study (up to 4years/5 to 8 years/9 to 11 years/12 or more years)
		Monthly household income	Total income of all residents in home in month prior to administration of questionnaires (≤ US$284/US$285 to US$852/US$853 to US$2557/>US$2557)
		Age group	Age group of individual (35 to 39 years/40 to 44 years)
		Self-rated treatment need	Self-rated need for dental treatment at time of study (yes/no)
		Dental pain	Report of dental pain in previous six months (yes/no)
		Dental appointment in the previous 12 months	At least one dental appointment in the previous 12 months (no/yes)
		Dental services	Type of service used during the last dental appointment (private/public /healthcare plan, other)
2^nd^ level–Contextual	Structural SDH	Municipal Human Development Index– 2010 MHDI	Summarized measure of basic living conditions of a population centered on health, knowledge, standard of living/income based on municipal data (very low, low, medium [≤0.699]/high [0.700–0.799]/very high [≥0.800])
		Gini coefficient	Measure of deviation of distribution of wealth (or buying power) among individuals or families in a municipality based on perfectly equal distribution (distribution tertiles)
	Intermediate SDH	Fluoridated water supply	Condition of public water supply regarding water fluoridation (absence/ presence)
		Oral health coverage	Estimated population coverage by primary oral health teams (< 50%/≥ 50%)

Monthly household income was determined in Brazilian currency and converted to US dollars (mean exchange rate in 2010: R$1.76 = US$1.00). Age group is a biological factor, which, together with the aforementioned individual factors, integrates the social determinants contained in the last two columns of the WHO theoretical model of the SDH [19] (S1 Fig, Final form of the Commission on Social Determinants of Health [CSDH] conceptual framework). Self-rated treatment need (yes/no) and dental pain in the previous six month (yes/no) express oral health-related biological factors. The two variables regarding dental appointments and dental services (at least one dental appointment in the previous 12 months [no/yes]; type of service used during the last dental appointment [private/public/healthcare plan, other) integrate factors regarding oral healthcare services, which also exert an impact on oral health [21]. Individuals who never visited a dentist were included in the “no” category of ‘dental appointment in the previous 12 months’ and were excluded as invalid data regarding the ‘dental services’ variable.

Contextual factors represent the social policies and wellbeing category in the first column of the WHO theoretical model of the SDH, which addresses structural determinants that intensely affect health by generating differences in power, prestige and access to essential resources [19]. Among such factors is included the Brazilian 2010 Municipal Human Development Index (2010 MHDI) which follows the same calculation and dimensions as the global Human Development Index. Up to 0.499, the MHDI is classified as very low and, rising from this figure, the classifications are denominated low, middle, high and very high with each increase of 0.100. An MHDI above 0.8 is classified as very high [25]. This classification was adopted in the present study to categorize the 2010 MHDI. However, as the three initial categories represented only 15% of the total sample, these categories were pooled into a single category (very low, low and medium).

The Gini income coefficient measures the difference in the distribution of income (or buying power) among individuals or families within a municipality based on a perfectly equal distribution. Thus, the coefficient ranges from zero (absolute equality) to 1 (absolute inequality) [26] and the categories were defined by distribution tertiles. These two indices were consulted in the 2013 Brazil Atlas of Human Development [27], which allows a selection based on data extracted from the 2010 demographic census. Fluoridated water supply is another contextual factor evaluated which is considered the broadest-scoped and most socially fair form of access to fluoride [28]. The National Basic Sanitation Survey performed by the IBGE in 2008 [29] was the source of information regarding the fluoridation of water in each municipality (absent/present). The last contextual factor evaluated was the estimated coverage of the resident population by primary care oral health teams (oral health coverage), which corresponds to the mean monthly number of primary care oral health teams for every 3000 individuals in relation to the total population of the municipality in the year analyzed. Greater coverage by primary care oral health teams indicated a greater potential offer of and access to basic dental services. The BMH establishes 50% of oral health coverage as the parameter to be achieved by municipalities, which was used as the cutoff point for this variable. The webpage of the Performance Index of the Brazilian Public Healthcare System was consulted to acquire data for 2010 regarding each municipality [30].

**Fig 1:**
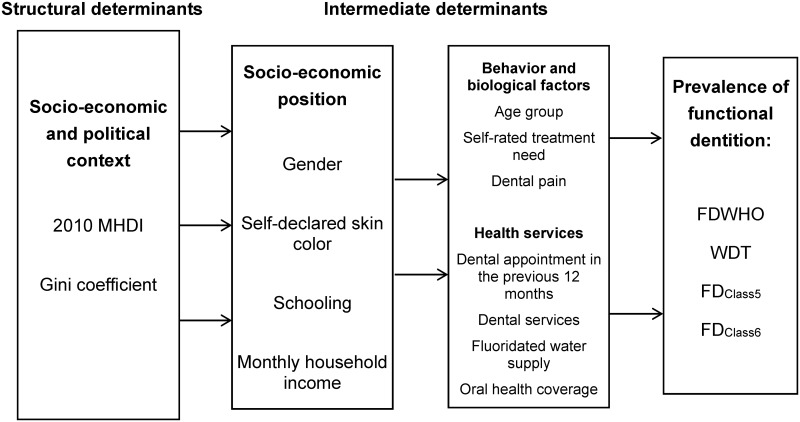
Conceptual model for the influence of Social Determinants of Health on functional dentition. Abbreviations: FDWHO: the World Health Organization functional dentition concept; WDT: the well-distributed teeth concept; FDClass5: functional dentition classified by esthetics and occlusion; FDClass6: functional dentition classified by esthetics, occlusion and periodontal status.

A theoretical model was created (Fig 1) to explain the influence of these factors on the four outcomes studied, which was based on reference models [19,21] and was used to guide the statistical analyses and interpretation of the findings.

### Statistical analysis

Descriptive statistics were first performed to determine the distribution of the sample with regard to oral health status, individual and contextual factors. The prevalence rates of the outcomes of each category of independent variables and respective 95% confidence intervals (CIs) were calculated. These analyses were weighted by the sample weight to account for the design effect of complex sampling using the *Complex Samples* command of the SPSS software. The sample weights were calculated by the inverse of the probability equations (f)^-1^ (in which f = n/N) and added to the files of the individuals examined. Detailed information on weighting can be found in other publication [15]. Next, multilevel (two levels) mixed-effect Poisson regression analyses were performed to determine the effect of the individual factors (first level) as well as the influence of municipal context (second level) on the distribution of the outcomes. The first step of the analysis involved the evaluation of the effect of each level (individual and contextual) through the calculation of unadjusted prevalence ratios (PR) and respective 95% CIs. The reference category for all the independent variables was that which corresponded to the worst condition. The multiple regression analyses began with the random intercept model (null model) to determine whether the effect of the context (municipalities) was significant. For such, variance among the municipalities was analyzed and the Likelihood Ratio (LR) test was performed.

Next, multiple, multilevel, mixed-effects models were created based on the entry criteria of p ≤ 0.2 for the independent variables. This value was adopted with the aim of minimizing residual confounding due to the risk of omitting relevant variables (potential confounders) for FD in the models [31]. First, the individual factors were incorporated into the null model, followed by the contextual factors. Next, variables with no significant association with the outcomes in the multiple models, but had been selected in the bivariate analyses were re-incorporated one-by-one to the multiple models to test their associations with each outcome and their contribution to the fit of the model. The maintenance of variables in the final models was determined by statistical significance (p≤0.05) as well as by the best fit of the multiple model, which was evaluated based on deviance, variance on the contextual level (municipalities) and the LR test. The mixed-effects models were generated by the ‘xtmepoisson’ command on STATA software. In order to compare the results for the four outcomes according to the independent variables, the adjusted PRs and respective 95% CIs were plotted in a graph. All statistical analyses were performed using SPSS^®^ 17.0 (SPSS Inc., Chicago, IL, USA) and STATA^®^ 12.0 (StataCorp, College Station, Texas, USA) programs, and graph was generated on Microsoft Excel^®^ 2013.

### Ethical aspects

The Brazilian National Human Research Ethics Committee approved the 2010 NOHS under process number 15498 on July 1^st^, 2010. Written informed consent was obtained from all participants.

## Results

The initial sample identified for participation in the survey consisted of 9779 adults, but examinations were not performed on 215, resulting in a final sample of 9564 adults. The majority was female (63.4%), was brown or white (87.5%) and earned a monthly household income from US$285 to US$852 (53.4%). Mean schooling was 8.5 years (95% CI: 8.1–8.9). The mean number of sound teeth and mean DMFT index were 13.6 (95% CI: 13.0–14.1) and 16.7 (95% CI: 16.2–17.3), respectively. The means of the missing and filled components were around 7.4. A total of 1039 adults were edentulous in the mandible and/or maxilla (10.1%; 95% CI: 8.5–12.0%). The highest CPI codes encountered among the sextants were excluded, calculus and sound in 31.7% (95% CI: 29.0–34.6%), 28.4% (95% CI: 25.7%-31.1%) and 17.9% (95% CI: 15.8–20.2%) of the sample, respectively. Regarding clinical attachment loss, the highest CAL codes encountered among the sextants were attachment loss of 0–3 mm and excluded in 51.0% (95% CI: 47.6–54.4%) and 29.8% (95% CI: 27.2–32.5%) of the total sample, respectively.

The prevalence rates of FDWHO and WDT were 77.9% (95% CI: 75.4 to 80.2) and 72.9% (95% CI: 70.1 to 75.4), respectively. When FD_Class5_ and FD_Class6_ were considered, however, the prevalence rates were the lowest: 42.6% (95% CI: 40.0 to 45.2%) and 40.3% (95% CI: 37.7 to 43.0%), respectively (Table 2). Among the individual factors, more accentuated differences were found among the categories of variables regarding educational and economic aspects. A typical social gradient was identified, in which individuals with a higher level of schooling and household income had greater prevalence rates of the four outcomes. The differences in the degree of schooling were more pronounced in relation to FD_Class5_ and FD_Class6_. The prevalence of adults with 12 or more years of study (≈ 64%) were nearly threefold greater than that of those with only up to four years of study. A similar result was found regarding monthly household income, as adults with an income >US$2557 had a 2 to 2.5-fold greater prevalence of FD_Class5_ and FD_Class6_ in comparison to those with an income ≤US$284. On the second level (municipal), the 2010 MHDI was the only variable that demonstrated significant differences (p ≤ 0.05) among its categories for all outcomes. (Table 2).

**Table 2:** Descriptive analyses of individual and contextual factors associated with four oral health outcomes among 9,564 Brazilian adults, 2010.

			Oral health outcomes								
				1 –FDWHO		2 –WDT		3 –FD_Class5_		4 –FD_Class6_^h^	
Level classification	Variables	Categories	n	%	95% CI	%	95% CI	%	95% CI	%	95% CI
	Prevalence rates	Yes	-	77.9	75.4;80.2	72.9	70.1;75.4	42.6	40.0;45.2	40.3	37.7;43.0
		No	-	22.1	19.8;24.6	27.1	24.6;29.9	57.4	54.8;60.0	58.5	55.8;61.1
Individual factors	Gender^a^	Female	6,287	75.3	72.1;78.2	70.3	67.1;73.4	41.7	38.6;44.9	40.1	36.8;43.4
		Male	3,277	**82.4**	**79.3;85.2**	**77.3**	**73.5;80.6**	**44.0**	**39.9;48.2**	**42.1**	**37.9;46.4**
	Self-declared skin color^a^	Black	1,002	73.5	66.8;79.3	67.7	60.8;73.8	42.5	35.8;49.4	38.1	32.1;44.5
		Brown	4,280	75.9	72.1;79.3	70.5	66.6;74.2	36.9	33.8;40.1	35.4	32.4;38.6
		White	4,049	80.3	77.3;83.0	**75.7**	**72.3;78.9**	46.6	**42.8;50.4**	45.2	**41.4;49.1**
		Yellow	162	77.7	62.0;88.1	77.0	61.5;87.5	54.5	40.8;67.6	50.7	34.8;66.5
		Indigenous	71	78.6	61.0;89.6	70.3	49.0;85.3	48.7	27.7;70.2	48.7	27.7;70.2
	Schooling^b^	Up to4 years	1,633	61.6	55.8;67.0	54.6	49.3;59.8	24.2	20.7;28.1	21.5	17.7;25.8
		5 to 8 years	2,673	**73.2**	**69.4;76.7**	**66.6**	**62.5;70.4**	**35.1**	**30.6;39.9**	32.7	28.4;37.4
		9 to 11 years	2,963	**83.5**	**80.1;86.5**	**79.7**	**75.6;83.3**	**46.9**	**42.9;51.0**	**45.2**	**41.2;49.3**
		12 or more years	2,226	**92.4**	**89.3;94.6**	**90.2**	**86.7;92.8**	**64,7**	**59.0;70.1**	**64.4**	**58.6;69.7**
	Monthly householdincome^c^	≤US$284	1,404	67.7	62.2;72.7	60.2	54.0;66.1	26.7	21.4;32.7	21.8	17.2;27.2
		US$285—US$852	4,687	**75.3**	**72.2;78.2**	**69.8**	**66.5;72.9**	**37.4**	**34.7;40.3**	35.7	33.0;38.6
		US$853—US$2557	2,741	**84.8**	**81.4;87.7**	**81.2**	**77.4;84.4**	**56.0**	**51.0;60.8**	**55.2**	**50.1;60.2**
		>US$2557	505	**93.2**	**86.0;96.9**	**91.0**	**82.8;95.5**	**59.0**	**42.2;74.0**	**58.8**	**42.0;73.8**
	Age group^a^	40 to 44 years	4,537	70.4	67.3;73.4	64.7	61.3;68.0	33.1	28.9;37.5	31.7	27.5;36.2
		35 to 39 years	5,027	**84.7**	**81.8;87.3**	**80.3**	**76.9;83.3**	**51.3**	**47.8;54.8**	49.1	45.3;53.0
	Self-rated treatment need^d^	Yes	7,360	79.2	76.7;81.5	73.5	70.7;76.1	39.8	37.4;42.2	37.6	35.2;40.1
		No	1,999	74.6	69.1;79.4	71.9	66.3;77.0	51.4	45.1;57.6	**50.8**	**44.5;57.2**
	Dental pain^e^	Yes	2,344	79.2	75.9;82.1	72.2	68.3;75.7	36.6	32.3;41.1	34.6	30.7;38.9
		No	7,151	**77.7**	**74.7;80.3**	**73.4**	**70.3;76.3**	45.1	41.6;48.5	**43.3**	**39.6;47.0**
	Dental appointment in the previous 12 months^f^	No	4,965	75.2	71.8;78.3	69.6	66.1;72.8	40.5	37.5;43.6	38.9	35.9;42.1
		Yes	4,446	**81.4**	**78.4;84.0**	**77.1**	**73.7;80.2**	**45.2**	**40.7;49.8**	**43.2**	**38.7;47.8**
	Dental services^g^	Private	3,901	80.3	77.3;83.0	76.1	72.6;79.2	48.1	44.2;52.0	46.9	43.0;50.8
		Public	3,524	**73.7**	**69.8;77.2**	**68.3**	**64.4;72.1**	**35.1**	**31.2;39.3**	**32.6**	**29.1;36.4**
		Health plan, other	1,387	**87.8**	**83.1;91.3**	**81.4**	**76.1;85.8**	**47.9**	**41.2;54.8**	**46.1**	**39.3;53.0**
Contextual factors	2010 MHDI^a^	Very low, low, medium (≤0.699)	1,171	67.5	60.4;73.9	62.4	54.1;69.9	29.4	23.4;36.2	28.2	22.2;35.1
		High (0.700–0.799)	6,239	**77.6**	**74.4;80.5**	**71.7**	**68.6;74.5**	**43.6**	**40.5;46.7**	**41.8**	**38.6;45.1**
		Very high (≥0.800)	2,154	**84.5**	**80.5;87.9**	**81.6**	**77.1;85.4**	**47.9**	**42.2;53.8**	**46.1**	**39.5;52.7**
	Gini coefficient^a^	Third tertile (greatest inequality)	3,699	76.7	74.4;78.8	71.2	68.6;73.7	39.3	36.1;42.6	38.4	35.3;41.6
		Second tertile	2,465	81.8	74.5;87.3	77.2	70.6;82.8	47.8	42.6;53.1	46.7	41.0;52.5
		First tertile	3,400	77.8	74.3;80.9	72.9	69.0;76.4	43.2	39.6;46.8	40.9	37.2;44.8
	Fluoridated water supply^a^	Absent	2,061	70.7	65.1;75.8	64.6	58.1;70.6	36.1	28.8;44.0	33.5	26.4;41.5
		Present	7,503	**78.9**	**76.3;81.3**	**74.0**	**71.2;76.7**	**43.5**	**40.7;46.4**	41.8	38.9;44.8
	Oral health coverage^a^	< 50%	7,673	78.8	76.2;81.3	74.3	71.2;77.1	43.5	40.5;46.5	41.9	38.7;45.1
		≥ 50%	1,891	74.9	68.2;80.6	**68.6**	**62.2;74.3**	39.9	34.6;45.3	**37.6**	**32.1;43.5**

Abbreviations: FDWHO: the World Health Organization functional dentition concept; WDT: the well-distributed teeth concept; FD_Class5_: functional dentition classified by esthetics and occlusion; FD_Class6_: functional dentition classified by esthetics, occlusion and periodontal status; CI: confidence interval.

^a^ No missing data;

^b^ 69 (0.7%) missing data;

^c^ 227 (2.3%) missing data;

^d^ 205 (2.2%) missing data;

^e^ 69 (0.7%) missing data;

^f^ 153 (1.1%) missing data;

^g^ 672 never visited a dentist + 80 missing data (7.3%);

^h^ 172 (1.2%) missing periodontal exams; values in bold type indicate p ≤ 0.05 in bivariate analysis

In final multiple models, among the variables representative of socio-demographic and socioeconomic position, higher prevalence rates of the four outcomes were found for the male gender, adults with a higher level of schooling and those with a higher monthly household income (Table 3). Associations of greater magnitude were found between individuals with 12 or more years of schooling and the four outcomes: FDWHO (PR: 1.41; CI 95%: 1.29 to1.55), WDT (PR: 1.58; CI 95%: 1.43 to 1.74), FD_Class5_ (PR: 2.03; CI 95%: 1.77 to 2.32) and FD_Class6_ (PR: 2.15; CI 95%: 1.87 to 2.47). Self-declared skin color demonstrated a significant difference in the brown category (PR: 0.87; CI 95%: 0.78 to 0.98) in comparison to the black category only for FD_Class5_. Among the variables representative of biological factors, age group was significantly associated with the four outcomes. Higher prevalence rates were found for those in the youngest age group. Also in this group of factors, self-rated treatment need was associated with the outcomes in different directions. The prevalence of FDWHO was lower among adults who reported not having treatment needs (PR: 0.93; CI 95%: 0.87 to 0.99) in comparison to those who reported having treatment needs. Moreover, a positive association was found between a lack of self-rated treatment need and both FD_Class5_ and FD_Class6_ (PR: 1.11 and 1.13, respectively).

**Table 3:** Unadjusted and adjusted multilevel mixed-effect Poisson regression analyses of individual and contextual factors associated with four oral health outcomes among Brazilian adults, 2010.

			Oral health outcomes															
			1 –FDWHO				2 –WDT				3 –FD_Class5_				4 –FD_Class6_			
Level classification	Variables^a^	Categories	PR^un^	95% CI	PR^ad^ ^b^	95% CI	PR^un^	95% CI	PR^ad^ ^b^	95% CI	PR^un^	95% CI	PR^ad^	95% CI	PR^un^	95% CI	PR^ad^	95% CI
Individual factors	Gender	Female	Ref.		Ref.		Ref.		Ref.		Ref.		Ref.		Ref.		Ref.	
		Male	**1.08**	1.03;1.14	**1.10**	1.04;1.16	**1.10**	1.04;1.16	**1.12**	1.06;1.18	**1.15**	1.08;1.23	**1.17**	1.09;1.25	**1.14**	1.07;1.23	**1.15**	1.08;1.24
	Self-declared skin color^a^	Black	-	-	-	-	Ref.		-	-	Ref.		Ref.		Ref.		-	-
		Brown	-	-	-	-	0.99	0.91;1.08	-	-	0.92	0.82;1.03	**0.87**	0.78;0.98	0.93	0.83;1.05	-	-
		White	-	-	-	-	**1.10**	1.01;1.20	-	-	**1.17**	1.05;1.31	0.98	0.87;1.10	**1.22**	1.08;.37	-	-
	Schooling	Up to4 years	Ref.		Ref.		Ref.		Ref.		Ref.		Ref.		Ref.		Ref.	
		5 to 8 years	**1.19**	1.10;1.28	**1.17**	1.07;1.27	**1.23**	1.13;1.34	**1.21**	1.11;1.33	**1.35**	1.19;1.54	**1.26**	1.11;1.44	**1.37**	1.20;1.56	**1.27**	1.11;1.45
		9 to 11 years	**1.36**	1.27;1.47	**1.29**	1.19;1.41	**1.50**	1.39;1.63	**1.43**	1.30;1.56	**1.87**	1.66;2.11	**1.64**	1.44;1.86	**1.93**	1.70;2.18	**1.67**	1.47;1.91
		12 or more years	**1.51**	1.40;1.63	**1.41**	1.29;1.55	**1.71**	1.57;1.86	**1.58**	1.43;1.74	**2.56**	2.27;2.89	**2.03**	1.77;2.32	**2.75**	2.43;3.12	**2.15**	1.87;2.47
	Monthly household income	≤US$284	Ref.		Ref.		Ref.		Ref.		Ref.		Ref.		Ref.		Ref.	
		US$285—US$852	**1,11**	1.03;1.20	1.05	0.97;1.14	**1.16**	1.07;1.25	1.08	0.99;1.17	**1.21**	1.08;1.36	1.04	0.92;1.17	**2.25**	1.11;1.41	1.08	0.96;1.22
		US$853—US$2557	**1.27**	1.18;1.37	**1.10**	1.01;1.21	**1.37**	1.26;1.49	**1.14**	1.04;1.26	**1.74**	1.56;1.96	**1.27**	1.11;1.44	**1.85**	1.64;2.09	**1.35**	1.18;1.53
		>US$2557	**1.39**	1.24;1.56	1.14	1.00;1.30	**1.55**	0.38;1.75	**1.19**	1.04;1.37	**2.35**	2.01;2.73	**1.48**	1.25;1.76	**2.53**	2.16;2.96	**1.57**	1.32;1.87
	Age group	40 to 44 years	Ref.		Ref.		Ref.		Ref.		Ref.		Ref.		Ref.		Ref.	
		35 to 39 years	**1.27**	1.21;1.32	**1.23**	1.17;1.30	**1.32**	1.25;1.38	**1.28**	1.21;1.34	**1.55**	1.45;1.66	**1.53**	1.42;1.64	**1.56**	1.46;1.68	**1.56**	1.45;1.67
	Self-rated treatment need^a^	Yes	Ref.		Ref.		-	-	-	-	Ref.		Ref.		Ref.		Ref.	
		No	0.98	0.97;1.00	**0.93**	0.87;0.99	-	-	**-**	-	0.99	0.95;1.04	**1.11**	1.02;1.20	**1.04**	0.99;1.08	**1.13**	1.04;1.23
	Dental appointment in the previous 12 months	No	Ref.		Ref.		Ref.		Ref.		Ref.		-	-	Ref.		-	-
		Yes	**1.13**	**1.08;1.18**	**1.06**	**1.01;1.12**	**1.14**	**1.08;1.19**	**1.06**	1.00;1.11	**1.11**	**1.04;1.19**	-	-	**1.13**	**1.05;1.21**	-	-
Contextual factors	2010 MHDI	Very low, low, medium (≤0.699)	Ref.		Ref.		Ref.		Ref.		Ref.		Ref.		Ref.		Ref.	
		High (0.700–0.799)	**1.18**	1.09;1.29	1.04	0.95;1.14	**1.22**	1.12;1.35	1.04	0.94;1.15	**1.46**	1.25;1.71	**1.17**	1.00;1.37	**1.47**	1.26;1.72	1.16	0.99;1.37
		Very high (≥0.800)	**1.37**	1.24;1.52	**1.13**	1.02;1.25	**1.49**	1.33;1.68	**1.18**	1.05;1.32	**1.94**	1.56;2.40	**1.41**	1.16;1.73	**1.97**	1.59;2.45	**1.42**	1.16;1.75
	Fluoridated water supply	Absent	Ref.		Ref.		Ref.		Ref.		Ref.		Ref.		Ref.		Ref.	
		Present	**1.21**	1.12;1.30	**1.18**	1.10;1.27	**1.24**	1.14;1.35	**1.21**	1.12;1.31	**1.36**	1.18;1.58	**1.20**	1.04;1.38	**1.37**	1.17;1.59	**1.22**	1.05;1.41

Abbreviations: FDWHO: the World Health Organization functional dentition concept; WDT: the well-distributed teeth concept; FD_Class5_: functional dentition classified by esthetics and occlusion; FD_Class6_: functional dentition classified by esthetics, occlusion and periodontal status; PR^un^ prevalence ratio unadjusted from bivariate analyses; PR^ad^ prevalence ratio adjusted final multiple model; CI: confidence interval; Ref.: Reference;

^a^ Self-declared skin color and self-rated treatment need not selected (p > 0.20) for multiple analysis of FDWHO and WDT, respectively;

^b^ values adjusted by dental services variable; values in bold type indicate p ≤ 0.05 in final multiple model

Dental appointment in the previous 12 months constituted the last individual factor associated with FDWHO and WDT. The prevalence of these two outcomes were higher among adults who reported having had a dental appointment in the previous 12 months (PR: 1.06) than in those who reported not having had a dental appointment in this period of time (Table 3).

**Fig 2:**
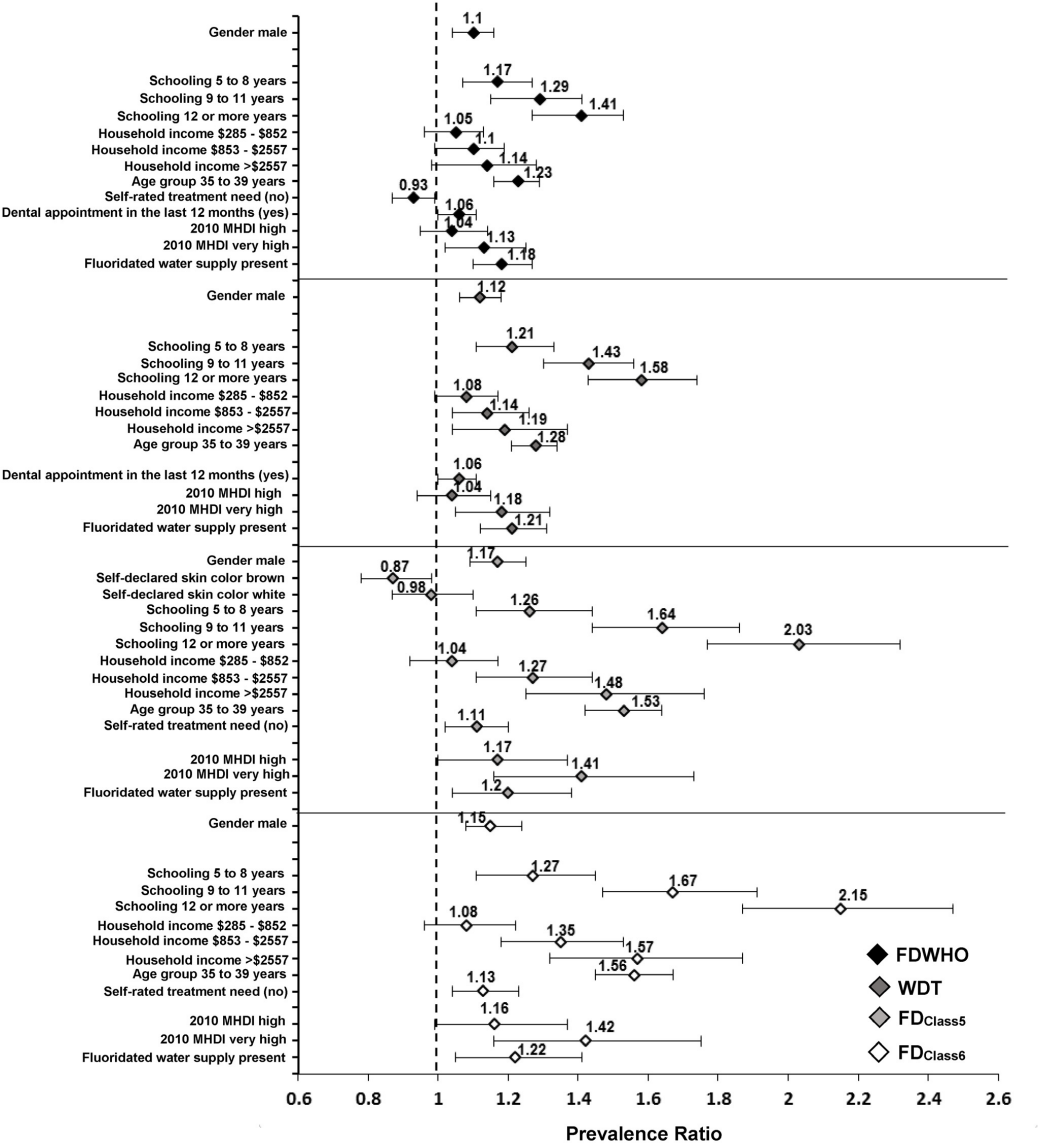
Adjusted Prevalence Ratios and respective 95% Confidence Intervals for the outcomes according to the independent variables. Abbreviations: FDWHO: the World Health Organization functional dentition concept; WDT: the well-distributed teeth concept; FDClass5: functional dentition classified by esthetics and occlusion; FDClass6: functional dentition classified by esthetics, occlusion and periodontal status.

Among the contextual factors on the second level, the 2010 MHDI and fluoridated water supply were maintained in the final models of the four outcomes, the prevalence rates of which were greater among adults in municipalities with a very high (> 0.8) 2010 MHDI (FDWHO—PR: 1.13; WDT—PR: 1.18; FD_Class5_ –PR: 1.41; FD_Class6_ –PR: 1.42) in comparison to those in municipalities with a very low, low and medium (≤ 0.699) 2010 MHDI. Fluoridated water supply also contributed to a greater prevalence of the four outcomes (Table 3). The greatest association magnitudes with individual and contextual factors were found for FD_Class5_ and FD_Class6_ (Fig 2).

Table 4 displays the parameters of the fixed and random effects in the null and multiple models of the four oral health outcomes. The pertinence of performing multilevel analysis was evaluated by the random intercept models (null models) generated for each outcome, which determined that the effect of the context was significant. FD_Class5_ and FD_Class6_ demonstrated the greatest variance among the municipalities (≥ 0.093), but all outcomes demonstrated significant results regarding the contextual effects (p < 0.05; LR test). Area variances were lower than individual level variances.FD_Class5_ and FD_Class6_ demonstrated the highest values of area variances in the final models in comparison with FDWHO and WDT and only these classes maintained significant p-values (< 0.001) in the LR test of the final models.

**Table 4:** Fixed and random effects parameters of multilevel mixed-effect Poisson regression analyses in the null and multiple models for the four oral health outcomes.

	Oral health outcomes											
	1 –FDWHO			2 –WDT			3 –FD_Class5_			4—FD_Class6_		
	Null Model	Model with Individual factors	Final Model	Null Model	Model with Individual factors	Final Model	Null Model	Model with Individual factors	Final Model	Null Model	Model with Individual factors	Final Model
Fixed effects												
Intercept	-0.33	-0.76	-0.92	-0.44	-1.00	-1.18	-1.08	-1.86	-2.10	-1.13	-2.06	-2.31
95% CI	-0.37;-0.29	-0.87;-0.65	-1.05;-0.78	-0.48;0.39	-1.12;-0.88	-1.32;-1.04	-1.15;-1.00	-2.04;-1.68	-2.32;-1.89	-1.21;-1.05	-2.22;-1.90	-2.51;-2.11
Random effects												
Municipal level												
Variance	0.011	0.007	<0.062^−10,000^	0.022	0.013	0.002	0.093	0.050	0.026	0.094	0.057	0.032
Standard Error	0.004	0.003	0.0016^−10,000^	0.006	0.005	0.002	0.025	0.016	0.012	0.025	0.017	0.013
LR test												
* χ*^2^	28.7	13.03	0.00	61.82	32.94	0.82	128.97	63.58	18.27	133.73	82.01	27.00
p	<0.001	<0.001	1.000	0.001	<0.001	0.182	<0.001	<0.001	<0.001	<0.001	<0.001	<0.001
AIC	18436.8	15963.0	15933.7	17994.2	15767.9	15736.7	14197.3	12690.0	12669.2	13636.3	12437.2	12416.1

Abbreviations: FDWHO: the World Health Organization functional dentition concept; WDT: the well-distributed teeth concept; FD_Class5_: functional dentition classified by esthetics and occlusion; FD_Class6_: functional dentition classified by esthetics, occlusion and periodontal status; LR: likelihood ratio; CI: confidence interval; x^2^: chi-square test; p: p-value; AIC: Akaike Information Criteria

## Discussion

The prevalence of functional dentition varied considerably among the four different definitions studied, with the lowest rate found for the new definitions employed in the present study: FD_Class5_ and FD_Class6_. The other two definitions, which are widely employed in the literature as the closest concepts of functional dentition [2–6], had statistically similar prevalence rates due to the coinciding 95% CIs. All four outcomes were affected by individual and contextual factors, which lends support to the theory of SDH in the theoretical model employed (Fig 1).

The evaluation of the associations between the four definitions of functional dentition and SDH was employed for the first time on a population of Brazilian adults in the present study. Despite this, it is important to stress that the data were obtained indirectly through a databank provided by the BMH and some important SDH were not addressed during the data collection process, such as behavioral habits, the effects of which have previously been associated with functional dentition [6], and aspects linked to social capital. Moreover, since it was not possible to clinically verify occlusal contact between the teeth, a POP was defined as a pair of antagonist teeth. Another limitation concerning clinical exams is that only index teeth were evaluated on periodontal exams and each individual was classified based on the highest CPI and CAL values encountered. Indeed, epidemiological studies with large samples, such as the present sample that was approximately three times larger than the samples in the other studies [7–9], generally employ this method, as proposed by the WHO [18], due to its greater viability.

Brazilian adults have generally experienced an improvement in their dental status, as measured by the increase in the prevalence of the WHO definition of functional dentition (≥ 20 teeth present). In 2003, the prevalence rate of this definition was 54% [17] and increased to 77.9% based on the estimations calculated in the present study. Despite the improvements in the oral health status of Brazilian adults identified by the reduction in missing teeth and increase in the number of sound and restored teeth between 1986 and 2010 [32], this figure is still below the WHO goal of 96% of adults aged 35 to 44 years with functional dentition [33]. Nonetheless, the figure is higher than that reported in previous studies involving Brazilian adults [3] and similar to that reported for Vietnamese adults [7] as well as residents in urban areas of southeastern Brazil [6].

There are no previous studies involving the Brazilian population that have evaluated the effect of SDH on the outcomes WDT, FD_Class5_ and FD_Class6_. Thus, there are no national parameters for the purposes of comparison. However, comparing the present findings with those from international studies, the prevalence of WDT among Brazilian adults was similar to that reported for Vietnamese [7] and Chinese [34] adults (74% and 76%, respectively). A similar comparison can be made for FD_Class5_, which was present in 44% and 48% of Vietnamese [7] and Chinese [34] adults, respectively. With regard to FD_Class6_, no previous studies were found that have employed this definition. However, if the prevalence of periodontal disease in the populations analyzed by Nguyen *et al*. [7] and Zhang *et al*. [34] was similar to that found among Brazilian adults, it would be possible for the prevalence of FD_Class6_ to be similar also.

Individual factors, which were previously associated with functional dentition in adults [3–6], remained in the final multiple models of the four outcomes. Higher prevalence rates of these outcomes were found in adults of the male gender, those with a high level of schooling and household income and those in the lower age group. Poorer oral health status among women is commonly mentioned in studies [3–6,35], despite the lack of consensus [32] and the fact that it is not possile to explain this difference in biological terms. Indeed, gender is seen in SDH modles [19,21] as a factor that exerts an influence on socioeconomic position due to prejudices and discrimination, which leads to differences in the exposure and risk of intermediate determinants. Although gender inequalities are recognized, their consequences regarding oral health cannot yet be definitively determined. Evaluating the need for dental treatment among adults, Roncalli *et al*. [22] found no significant differences in the magnitude of needs between men and women, although the needs for restorative treatment and dental extraction were slightly higher among men. Thus, the gender differences observed may be explained by the effects of the social gradient in health [36].

The effects of schooling and monthly household income on the prevalence rates of the four outcomes follows the same association direction as the WHO definition of functional dentition [3,5,6], dental caries [23], missing teeth [4], periodontal disease [24] and the need for dental treatment [22]. A greater degree of schooling and higher monthly household income reflect a better oral health status. The social gradient is recognized as an important issue regarding systemic and oral heath [36] due to the mechanisms of social stratification and the establishment of social inequalities [19]. The situation is more aggravating when it is identified that less privileged individuals require more tooth extractions [22]. Thus, socioeconomic status affected FD_Class5_ and FD_Class6_ with greater magnitude than that found for FDWHO and WDT, especially with regard to schooling (Fig 2).

This situation leads to the reflection that whether the WHO is not increasing oral health inequalities in a biased manner by stipulating a global goal based merely on a quantitative criterion of functional dentition. Public health policies seek to achieve for everyone the retention of a functional dentition of at least 20 teeth in the mouth without consideration of the distribution or condition of these teeth, while only the most privileged individuals manage to retain a dentition that encompasses all the requirements of functionality in terms of esthetics, occlusion and periodontal status. Small increases in household income (US$285 to US$852) have not been sufficient to demonstrate significant increase in the prevalence rate of FD_Class5_ and FD_Class6_ with regard to the reference category. Another individual factor (age group), despite being narrow, was capable of identifying differences in the prevalence rates of the four outcomes. Younger adults (35 to 39 years) exhibited higher prevalence rates than older adults (40 to 44 years). Similar findings have been reported in previous studies [3–6], which is conceivable due to the cumulative effect of dental caries and the increase in the prevalence of periodontal disease with the increase in age, which lead to a lower retention of teeth.

Among the socio-demographic factors, self-declared skin color was significantly associated with FD_Class5_, as a worse condition was found among adults who declared having brown skin in comparison to those who declared having black skin. Racial inequalities are known to be more expressive among individuals of African descent [36] in comparison to whites. However, differences between those with black and brown skin were found with regard to the absence of functional dentition [4] as well as periodontal disease [24], but in opposite directions. Self-declared black Brazilian adults had a higher prevalence rate of absent functional dentition than self-declared brown adults [4], but this association was lost in the final model adjusted for schooling. In the case of periodontal disease, periodontal status has been found to be less favorable among those with brown skin than those with black skin [24], which is in agreement with the present findings. The effect of ethnicity on health conditions is influenced by a set of factors. A study on tooth loss identified that the material conditions of life and educational levels were the most important factors [37]. Thus, it is necessary to investigate this difference between individuals with brown and black skin color in greater detail and evaluate whether Brazilian program of the quotas for blacks in universities (implanted in the last decade) may have also benefitted their health, as higher levels of schooling are commonly associated with a better health status [3–6,23,24,36].

Self-rated treatment need was one of the representative behavioral and biological factors associated with FDWHO, FD_Class5_ and FD_Class6_. However, the association was also in different directions. The prevalence of FDWHO was lower among adults who reported no treatment needs in comparison to those who reported needs. As this is a subjective measure (self-rated), such differences may mean that, although FDWHO is one of the most used concepts of functional dentition in the literature [2–6], adults do not feel satisfied with this oral condition alone. In contrast, the effect of FD_Class5_ and FD_Class6_ on satisfaction with oral health seems to be more positive, as a greater prevalence rates of these outcomes were found among adults who reported not needing treatment. This may be due to the fact that such outcomes are more complete concepts of functional dentition, which favors a lower perception of treatment need or even the absence of need. However, further studies should be conducted to address subjective aspects related to the impact of the different outcomes on the quality of life of adults, which would allow more conclusive evaluations.

In the health services group of determinants, dental appointment in the previous 12 months was maintained in the final models of FDWHO and WDT. Having had at least one dental appointment in this period was associated with greater prevalence rates of these outcomes, which is in agreement with data from a study conducted in southern Brazil [3]. However, the same did not occur for FD_Class5_ and FD_Class6_ in the presence of other factors in the final multiple models. These oral conditions have broader-scoped requirements than the mere number of teeth in the mouth, such as the location of the teeth, occlusal contacts and periodontal status. Thus, one may expect the dependence on more complex treatments for their preservation, which may not be offered during dental appointments at primary healthcare services and are more restricted in terms of access as well as dependent on the existence of specialized dental centers [38]. The predominance of mutilating treatment (extraction) over conservative treatment is part of the recent history of public oral health in Brazil. While this situation has undergone positive transformations [28], it may not yet have been able to alter the profile of oral health status among Brazilian adults. The less conservative profile of dental services in developing nations has been noted internationally [35].

The variance of the four outcomes among the municipalities was significant in the tests performed (Table 4) and the outcomes remained associated with contextual factors in the final models, with greater magnitudes identified with regard to FD_Class5_ and FD_Class6_. Adults from municipalities with a very high 2010 MHDI (≥ 0.800) and fluoridated water supply had higher prevalence rates of the four outcomes in comparison to adults from municipalities with a very low, low and medium 2010 MHDI (≤ 0;699) and no fluoridation of the public water supply. The effect of such structural determinants on functional dentition was also found in an adult population in southern Brazil [3]. Investigating the influence of context on needs for dental treatment, Roncalli *et al*. [22] found that higher MHDI values were associated with lower needs for dental treatment. Locations with higher MHDI have more favorable living conditions, a better quality of life and possibly better access to more qualified, conservative oral health services. Thus, albeit in an indirect fashion, a favorable context of a municipality can exert a positive influence on the retention of teeth in adults. The effects of the fluoridation of public water supplies may be explained by its capacity to reduce the prevalence and incidence of dental caries [39], which is the main cause of missing teeth [4] and, consequently, the lower degree of tooth retention. However, the interpretation of the effect of this variable on the outcomes is somewhat limited in the present study due to the fact that there is no available data to determine how long the municipality had a fluoridated water supply. Nonetheless, this variable may be used as a *proxy* for better organized municipal oral health services.

Despite the significant associations among these contextual factors and the four outcomes, area variances were lower than individual level variances, which reflects a greater influence of individual rather than contextual factors on the occurrence of the outcomes. This difference was less striking for FD_Class5_ and FD_Class6_, which suggests a greater influence of municipal contexts in more complete, broader-scoped concepts of functional dentition.

## Conclusions

The incorporation of the functionality criteria of new definitions of functional dentition that considers esthetics, occlusion and periodontal status considerably restricted the prevalence of this condition among adults. Moreover, striking inequalities were found on the individual and contextual levels regarding the four definitions studied, which need to be addressed in intersectoral actions. The significant effect of individual factors and context on oral health outcomes demonstrates the need to connect the approach focused on individual behavior and the inclusion of structural determinants due to their causal priority.

## Supporting Information

S1 Fig Final form of the Commission on Social Determinants of Health (CSDH) conceptual framework. Font: Solar O, Irwin A. A conceptual framework for action on the social determinants of health. Social Determinants of Health Discussion. Geneva: World Health Organization; 2010, p. 6.
(PDF)
