# Supplementary material for: Functional Dentition in Brazilian Adults: An Investigation of Social Determinants of Health (SDH) Using a Multilevel Approach
Source: PLoS One. 2016 Feb 10;11(2):e0148859. doi: 10.1371/journal.pone.0148859 (PMC4749636; doi:10.1371/journal.pone.0148859)

S1 Figure. Final form of the Comission on Social Determinants of Health (CSDH) conceptual framework. Font: Solar O, Irwin A. A conceptual framework for action on the social determinants of health. Social Determinants of Health Discussion. Geneva: World Health Organization; 2010, p. 6.

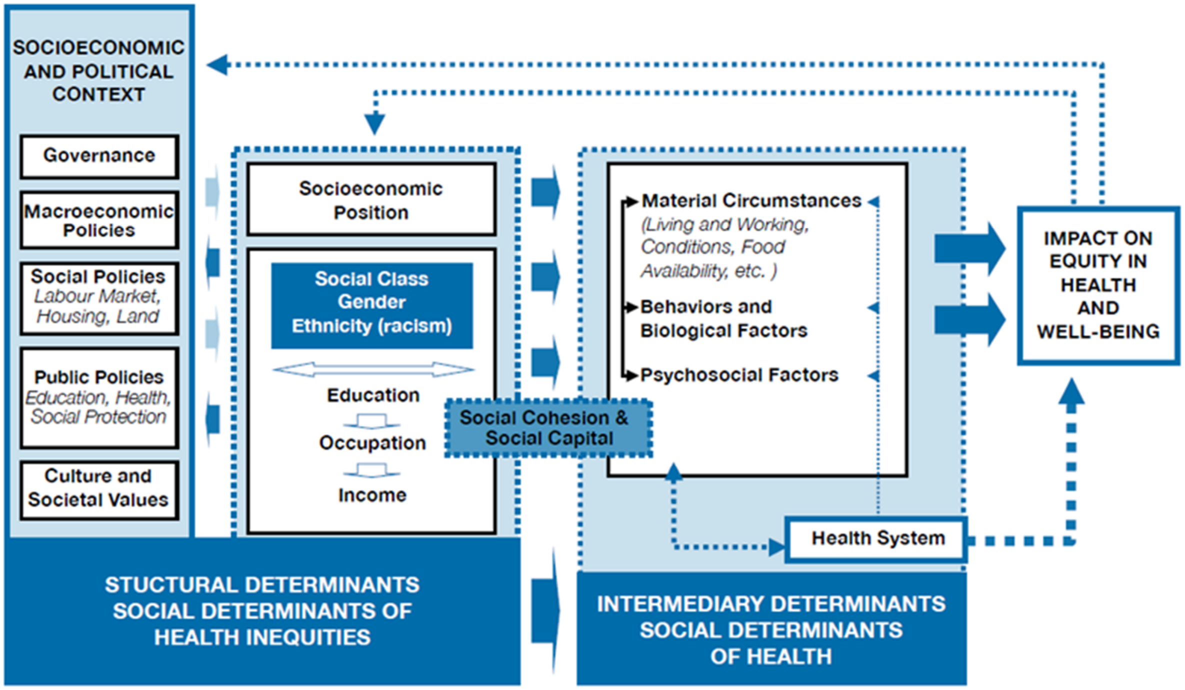

Supplement: S1 Fig — Font: Solar O, Irwin A. A conceptual framework for action on the social determinants of health. Social Determinants of Health Discussion. Geneva: World Health Organization; 2010, p. 6. (PDF) [file pone.0148859.s001.pdf]
